# Supplementary material for: Estimation-uncertainty affects decisions with and without learning opportunities
Source: Nat Commun. 2025 Jul 21;16:6706. doi: 10.1038/s41467-025-61960-2 (PMC12280070; doi:10.1038/s41467-025-61960-2)
Supplement: Supplementary file 1 — Supplementary Information [file 41467_2025_61960_MOESM1_ESM.pdf]

Supplementary information

Estimation-uncertainty affects decisions with and without learning opportunities

Kristoffer C. Aberg[1], Levi Antle[1], Rony Paz[1]

Supplementary Table 1. Outcome probabilities for the different conditions in Experiment 1.

Condition ($p_{Appetitive}$)	Outcome probability				Summed outcome probabilities per option						Expected value (EV)[1]		Shannon Entropy (H)[2]	
	Positive feedback		Negative feedback		Good option			Bad option			Good option	Bad option	Good option	Bad option
	+1₪	0₪	0₪	-1₪	+1₪	0₪	-1₪	+1₪	0₪	-1₪				
1.0	1.0	0.0	1.0	0.0	0.75	0.25	0	0.25	0.75	0.0	0.75₪	0.25₪	0.5623	0.5623
0.75	0.75	0.25	0.75	0.25	0.5625	0.375	0.0625	0.1875	0.625	0.1875	0.50₪	0.0₪	0.8647	0.9215
0.5	0.5	0.5	0.5	0.5	0.375	0.5	0.125	0.125	0.5	0.375	0.25₪	-0.25₪	0.9743	0.9743
0.25	0.25	0.75	0.25	0.75	0.1875	0.625	0.1875	0.0625	0.375	0.5625	0.0₪	-0.50₪	0.9215	0.8647
0.0	0.0	1.0	0.0	1.0	0	0.75	0.25	0	0.25	0.75	-0.25₪	-0.75₪	0.5623	0.5623

[1]The expected value (EV) is calculated as:

$EV = p_{PositiveFB}*(1\text{₪}*p_{Appetitive} + 0\text{₪}*p_{Avoid}) + p_{NegativeFB}*(0\text{₪}*p_{Appetitive} + -1\text{₪}*p_{Aversive})$

Where $p_{PositiveFB}$=0.75/0.25 for Good/Bad options and $p_{NegativeFB}=1-p_{PositiveFB}$.

[2]The Shannon entropy is calculated as:

$H=-\Sigma_i[p_i*\log(p_i)]$, where p_i is the outcome probability for outcome *i*.

Supplementary Table 2. Average learning performance versus chance-level for conditions in Experiment 1.

Condition ($p_{Appetitive}$)	*t*	*df*	Uncorrected p-value	Cohen's d	95% Confidence interval
1.0	10.870	49	<0.001	1.537	0.707 0.801
0.75	10.578	49	<0.001	1.500	0.660 0.746
0.5	6.689	49	<0.001	0.946	0.607 0.699
0.25	9.422	49	<0.001	1.333	0.646 0.715
0.0	10.886	49	<0.001	1.540	0.635 0.696

t is the t-statistics, df is the degrees of freedom, and uncorrected p-values are provided by performing two-tailed *t*-tests versus zero. Cohen's d estimates the effect size.

Supplementary Table 3. Pairwise comparisons for learning performance between conditions in Experiment 1.

Comparison	t	*df*	p-value	Cohen's d	95% confidence interval
0.0 vs 0.25	0.786	49	0.436	0.111	-0.053 0.023
0.0 vs 0.5	0.595	49	0.555	0.084	-0.030 0.056
0.0 vs 0.75	1.501	49	0.140	0.212	-0.087 0.013
**0.0 vs 1.0**	**3.605**	**49**	**<0.001**	**0.510**	**-0.138 -0.039**
0.25 vs 0.5	1.003	49	0.321	0.142	-0.028 0.083
0.25 vs 0.75	0.917	49	0.364	0.130	-0.071 0.027
0.25 vs 1.0	2.401	49	0.020	0.340	-0.135 -0.012
0.5 vs 0.75	1.871	49	0.067	0.265	-0.104 0.004
**0.5 vs 1.0**	**3.042**	**49**	**0.004**	**0.430**	**-0.168 -0.034**
0.75 vs 1.0	1.846	49	0.071	0.261	-0.107 0.005

t is the t-statistics, df is the degrees of freedom, and uncorrected p-values are provided by performing two-tailed *t*-tests. Cohen's d estimates the effect size. Comparisons marked by bold font survived Bonferroni-Holm correction for ten multiple comparisons (α=0.05/10=0.005).

Supplementary Table 4. Selection bias (collapsed across all comparisons) in the test phase of Experiment 1.

Predictor	Sum of Squares	*df*	Mean of Squares	F	p-value	η_p^2
**Good vs Good**						
(Intercept)	<0.001	1	<0.001	0.18	0.674	
Error	0.018	49	<0.001			
Condition	1.931	4	0.483	12.64	<0.001	0.205
Error(Condition)	7.490	196	0.038			
**Bad vs Bad**						
(Intercept)	<0.001	1	<0.001	2.73	0.105	

Error	0.011	49	<0.001			
Condition	0.805	4	0.201	6.02	<0.001	0.109
Error(Condition)	6.547	196	0.033			

F is the F-statistics for one-way repeated measures ANOVA with factor Condition, df is the degrees of freedom, and η_p^2 is the effect size (estimated by partial eta-squared).

Supplementary Table 5. Selection bias (pairwise comparisons) in the test phase of Experiment 1.

Predictor	Sum of Squares	*df*	Mean of Squares	F	p-value	η_p^2
(Intercept)	6.469	1	6.459	52.14	<0.001	
Error	6.070	49	0.124			
Comparison	1.582	9	0.176	3.37	<0.001	0.064
Error(Comparison)	22.987	441	0.052			
GvGBvB	0.500	1	0.500	4.36	0.042	0.082
Error(GvGBvB)	5.623	49	0.115			
Comparison x GvGBvB	0.740	9	0.082	1.31	0.232	0.026
Error(Comparison x GvGBvB)	27.787	441	0.063			

F is the F-statistics for two-way ANOVA with factor Comparison (10 comparisons) and GvGBvB (Good versus Good, Bad versus Bad), df is the degrees of freedom, and η_p^2 is the effect size (estimated by partial eta-squared).

Supplementary Note 1: Differences in decision strategies between the learning and the test phases

To test for consistent differences in decision strategies between the training and the test phases, two ANOVAs were conducted with one within-subject factor Phase (training, testing), one between-subject factor Experiment (the main experiment, the two experiments obtained from the online repository). The dependent variables in each ANOVA were the fitted decision weights for expected value (β_Q) and estimation uncertainty (β_U) for the Kalman:QU model.

Because model-fitted parameters scale with experimental parameters, e.g. β_Q scales with feedback magnitudes, all decision weights were standardized (z-scored) within each experiment prior to being added to the ANOVAs.

Standardized decision weights for expected value (β_Q) are shown in Supp. Fig. 1A. The corresponding ANOVA showed a main effect of Phase [F(1, 247)=20.2, p<0.001, η_p^2=0.076], but also a significant Phase x Experiment interaction [F(2, 247)=20.838, p<0.001, η_p^2=0.144, ANOVA]. Follow-up tests revealed significant differences between the training and test phases for the main experiment [t(49)=8.344, p<0.001, Cohens'd d=1.18], but not for the other two experiments [Dataset 1: t(99)=1.825, p=0.071, Cohens'd d=0.182; Dataset 2: t(99)=1.498, p=0.137, Cohens'd d=0.150]. One main difference between the experiments is that participants performed the main experiment in the lab, while data was collected in online experiments for the other two. The main differences in experimental parameters between experiments are shown below the x-axis in Supp. Fig. 1A.

Standardized decision weights for estimation uncertainty (β_U) are shown in Supp. Fig. 1B. The corresponding ANOVA showed a main effect of Phase [F(1, 247)=87.267, p<0.001, η_p^2=0.261], but also a significant Phase x Experiment interaction [F(2, 247)=23.254, p<0.001, η_p^2=0.159, ANOVA]. Follow-up tests revealed significant differences between the training and test phases for the main experiment [t(49)=9.678, p<0.001, Cohens'd d=1.369] and for one of the other two experiments [Dataset 1: t(99)=6.868, p=<0.001, Cohens'd d=0.687], but not for the other [Dataset 2: t(99)=0.350, p=0.727, Cohens'd d=0.035]. One main difference between the experiments is that the two experiments showing significant differences between the phases presented the pairs in an inter-leaved fashion during testing, while the experiment showing no effect presented pairs blockwise. The main differences in experimental parameters between experiments are shown below the x-axis in Supp. Fig. 1B. Future studies are clearly needed to confirm the interactions between decision strategies and experimental setups.

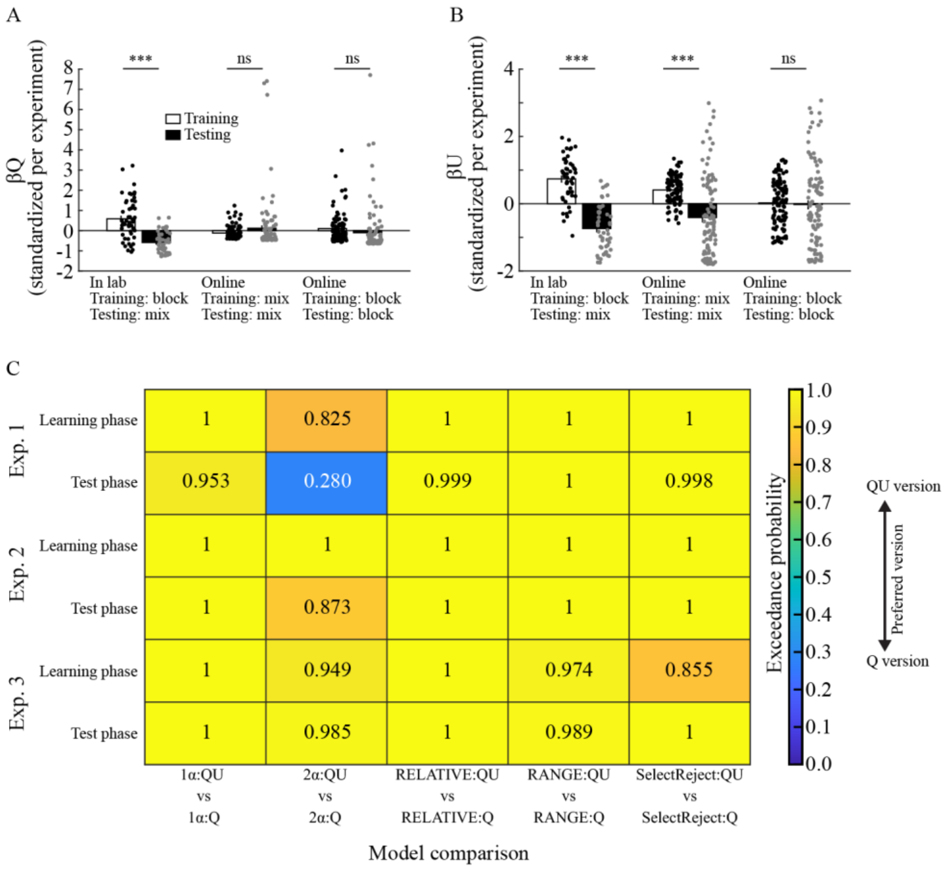

Supplementary Figure 1. Sample size n=50. **A.** Decision weight for expected values in each phase of each experiment. A significant difference between the training and the test phases was only observed for the experiment conducted in the lab (two-tailed *t*-tests; t(49)=8.344, p<0.001, Cohens'd d=1.18; t(99)=1.825, p=0.071, Cohens'd d=0.182; t(99)=1.498, p=0.137, Cohens'd d=0.150). **B.** Decision weight for estimation uncertainty in each phase of each experiment. Significant differences between the training and the test phases were observed for the experiments in which the test stimulus pairs were presented in an inter-leaved fashion (two-tailed *t*-tests; *t*(49)=9.678, p<0.001, Cohens'd d=1.369; *t*(99)=6.868, p<0.001, Cohens'd d=0.687; *t*(99)=0.350, p=0.727, Cohens'd d=0.035). **C.** Heat map displaying the protected exceedance probabilities for the pairwise cmparisons between a model with (X:QU) and without (X:Q) the estimation uncertainty component obtained from the Kalman-filter approach. The exceedance probabilities 0 and 1 indicate preferences for the X:Q and X:QU versions of the models, respectively. ***p<0.001, ns=not significant. All errorbars indicate the standard error of the mean.

Supplementary Note 2: Kalman-filter derived estimation uncertainty increases the performance of non-Kalman filter models

In the main analyses we show consistently that including estimation uncertainty as a decision parameter enhances model performance for models based on the Kalman-filter approach (i.e. Kalman:QU performs better than Kalman:Q). An even stronger statement can be made by showing similar contributions of

estimation uncertainty to non-Kalman filter models. Accordingly, we extracted estimation uncertainty estimates from the Kalman-filter approach (Equation 16-18 main text), and added them as decision parameters to all the non-Kalman filter models (Equation 21 main text). To demonstrate significant improvements in model fit, we then performed pairwise comparisons for each model type X (i.e. the original version without estimation-uncertainty X:Q and the new version with X:QU). This procedure was repeated for each of the five different non-Kalman filter models, and for each of the six separate instances (three experiments x two phases). The same HBI approach as in the main analyses was used to obtain the protected exceedance probability. Accordingly, if estimation uncertainty significantly contributes to model performance, we expect the X:QU model versions to provide a more parsimonious fit to behavior then their corresponding X:Q versions.

A heatmap showing the outcome of all pairwise model comparisons is displayed in Supp. Fig. 1C, with exceedance probabilities of 1/0 respectively indicating strong evidence in favor of X:QU/X:Q model versions. As can be seen in Supp. Fig. 1C, the X:QU model versions were preferred in 29/30 comparisons. Accordingly, because these results were highly consistent across all model types, experiments, and phases, they clearly demonstrate that estimation uncertainty needs to be considered when trying to understand human decision making in similar tasks.

Supplementary Note 3: Accounting for the magnitude effect during the learning phase

Across all experiments, Bavard et al. (2021)[1] reported a significant magnitude effect, i.e. overall performance was larger for high EV pairs, as compared to low EV pairs (see their paragraph entitled 'Overall correct response rate'). In our analyses, which separate each pair-by-pair comparison of the two experiments included in the main study, we observe higher performance for high EV pairs (i.e. AxBx pairs), as compared to low EV pairs (i.e. CxDx pairs). This was observed for both actual and model-fitted performances [all p-values<0.05; Supplementary Table 6]. In addition, we also demonstrate significant and positive correlations between actual- and model-fitted learning performance for all comparisons [all p-values<0.001; Supplementary Table 1]. Together, these results show that the model captures the magnitude effect in learning performance both on a group-level, as well as on the inter-individual level.

Supplementary Table 6. Differences in learning performance between high EV (A1B1; A2B2) pairs and low EV (C1D1; C2D2) pairs in terms of actual and model-fitted performance, and their correlations.

	Actual learning performance	Model learning performance	Correlations

	t	df	p-value	t	df	p-value	Pearson's r	p-value
Inter-mixed trials (Figure 3)								
A1B1 vs. C1D1	3.11	99	0.002	8.58	99	<0.001	0.73	<0.001
A2B2 vs. C1D1	3.61	99	<0.001	11.39	99	<0.001	0.75	<0.001
A1B1 vs. C2D2	3.38	99	0.001	8.05	99	<0.001	0.79	<0.001
A2B2 vs. C2D2	3.63	99	<0.001	9.81	99	<0.001	0.79	<0.001
Blocked trials (Figure 4)								
A1B1 vs. C1D1	2.80	99	0.006	8.49	99	<0.001	0.86	<0.001
A2B2 vs. C1D1	2.49	99	0.015	7.01	99	<0.001	0.87	<0.001
A1B1 vs. C2D2	2.15	99	0.034	7.72	99	<0.001	0.88	<0.001
A2B2 vs. C2D2	2.46	99	0.016	8.01	99	<0.001	0.84	<0.001

t is the t-statistics, df is the degrees of freedom, and uncorrected p-values are provided by performing two-tailed *t*-tests. All tests were two-tailed.

Supplementary Note 4: Model-based analysis of excluded experiments

The focus of the main study was on whether and how sampling-rates caused by partial feedback during learning affected subsequent decisions without feedback and no further learning. Two relevant datasets obtained from Bavard et al. (2001) were included and analyzed in the main study in an attempt to support the main results. Six other data sets provided by Bavard et al. (2001) were excluded because they could not be used to either support or weaken our main hypotheses. The reasons for exclusion were either because complete feedback was presented during learning (which removes the opportunity for sampling biases to arise) and/or because feedback was presented during the test phase (which confounds decision biases due to learning with those caused by, for example, exploration).

However, because our model-based approach provides a fine-grained analysis of behavior also in those datasets, they are analyzed here. Significant differences between our approach and the one used by Bavard et al. (2001) are listed below:

1. To our understanding, Bavard et al. (2021) fit their models across the eight different experiments. Yet, to check whether the ‘optimal’ model may differ between experiments, we fit the models to each experiment separately (as in our main study).

2. Bavard et al. (2021) first fit the parameters to the learning phase, and then use these parameters to also assess model-fits during the test phase. However, behavioral strategies may differ significantly during the learning and the test phase. In particular, participants explore to obtain information in conditions with feedback, something which affects value-based decision making and related model parameters. It is therefore unlikely that the same model parameters reflect behavior in conditions where feedback is available and where it is not (i.e. when no exploration occurs). To overcome this issue, we fit different parameters for the learning phase and for the subsequent test phase (as in our main study).

3. Similarly, participants may apply different behavioral strategies in conditions where feedback is available, as compared to conditions where no feedback is available. To overcome this issue, we allow for the learning models during the learning phase to differ from the subsequent test phase.

Models

As in the main study, the denotation of learning and decision models are separated by the colon symbol ‘:’. For example, a Kalman-filter learning model combined with a decision model which considers both expected values (Q) and uncertainty (U) is denoted by Kalman:QU. The types of models used for the different experimental conditions are detailed below.

Partial feedback conditions

For partial feedback conditions, the same six learning and the same two decision models as reported in the main study was utilized (see Equations 1-21, main text).

Complete feedback conditions

In complete feedback conditions, all of the learning models were updated to account for the additional information provided by the feedback for the rejected (non-selected) option, i.e. there is now a prediction error and a learning rate for both the selected and the rejected options:

$$Q(t+1)_{Selected} = Q(t)_{Selected} + \alpha_{Selected} * \delta(t)_{Selected} \quad \text{(S1)}$$

$$\delta(t)_{Selected} = R_{Selected} - Q(t)_{Selected} \quad \text{(S2)}$$

$$Q(t+1)_{Rejected} = Q(t)_{Rejected} + \alpha_{Rejected} * \delta(t)_{Rejected} \quad \text{(S3)}$$

$$\delta(t)_{Rejected} = R_{Rejected} - Q(t)_{Rejected} \quad \text{(S4)}$$

The '1α' model:

This model now updates both the selected and the rejected option in each trial according to Equations S1-S4.

The '2α' model:

This model now updates both the selected and the rejected option based on whether their outcomes are positive or negative prediction errors (Equation 3, main text).

The 'SelectReject' model:

Because an approximation for the prediction error of the rejected option is no longer needed, the 'SelectReject' model becomes identical to the new '1α' model and was therefore removed from the analyses.

The 'RELATIVE' model:

This model was updated in two ways. First, equation 9 of the main text is replaced by:

$$\delta(t)_c = \frac{(R_{Selected} + R_{Rejected})}{2} - V(t)_c \quad \text{(S5)}$$

Second, the prediction error of the rejected option is now calculated as:

$$\delta(t)_{Rejected} = R_{Rejected} - V(t)_c - Q(t)_{Rejected} \quad \text{(S6)}$$

The 'RANGE' model:

This model now updates also the expected value of the rejected option after scaling its feedback according to Equation 12 of the main text.

The 'Kalman' model:

This model now tracks the uncertainty for both options and updates respective learning rates according to Equations 16-17 of the main text.

Decision models:

For partial feedback conditions, there are two decision models, one based on expected value (denoted by ':Q'), and another which also considers uncertainty (i.e. ':QU'). However, because the uncertainty is equal for all options in complete feedback conditions, the ':QU' model was dropped in such conditions.

Model-fitting procedure

Learning phase

During learning, the model-fitting procedure is as described in the main text, i.e. the model-fits are determined by the similarity between actual- and model-fitted choices in the learning phase.

Test phase

In contrast to the main study, we now need to fit additional learning models for the experiments where feedback was presented during the test phase. We now also allow for different parameters to be fitted for the same model in the training and test phases, and for different learning models to explain behavior in the learning and test phases. Still, as in the main study, the model-fits are determined by the similarity between actual- and model-fitted choices in the test phase.

Results

The exceedance probabilities for the model-selection procedure are presented in Supplementary Figure 2. An immediate observation is that the 'optimal' model differs between experiments. The results grouped by feedback type during learning and testing are summarized below.

Learning with partial feedback and testing with partial feedback

For these conditions, the Kalman:QU model provides the best fit to behavior during the initial learning phase (Supplementary Fig. 2A,C) and the subsequent test phase (Supplementary Fig. 2B,D). While these results replicate the results of the main study, we also observe that the learning model which provides the most optimal input to the test phase are the two range-adaptation models (RELATIVE in Supplementary Fig. 2B; RANGE in Supplementary Fig. 2D).

Learning with complete feedback and testing with complete feedback

The 1α:Q model provides the best fit to the learning phase (Supplementary Fig. 2E,G), while the RELATIVE model provides the best fit to the test phase (Supplementary Fig. 2F,H).

Learning with complete feedback and testing with no feedback

The 1α:Q and RANGE:Q models provide the best fit to the learning phase (Supplementary Fig. 2I,K), while the RELATIVE and Kalman:Q models provide the best fit to the test phase (Supplementary Fig. 2J,L).

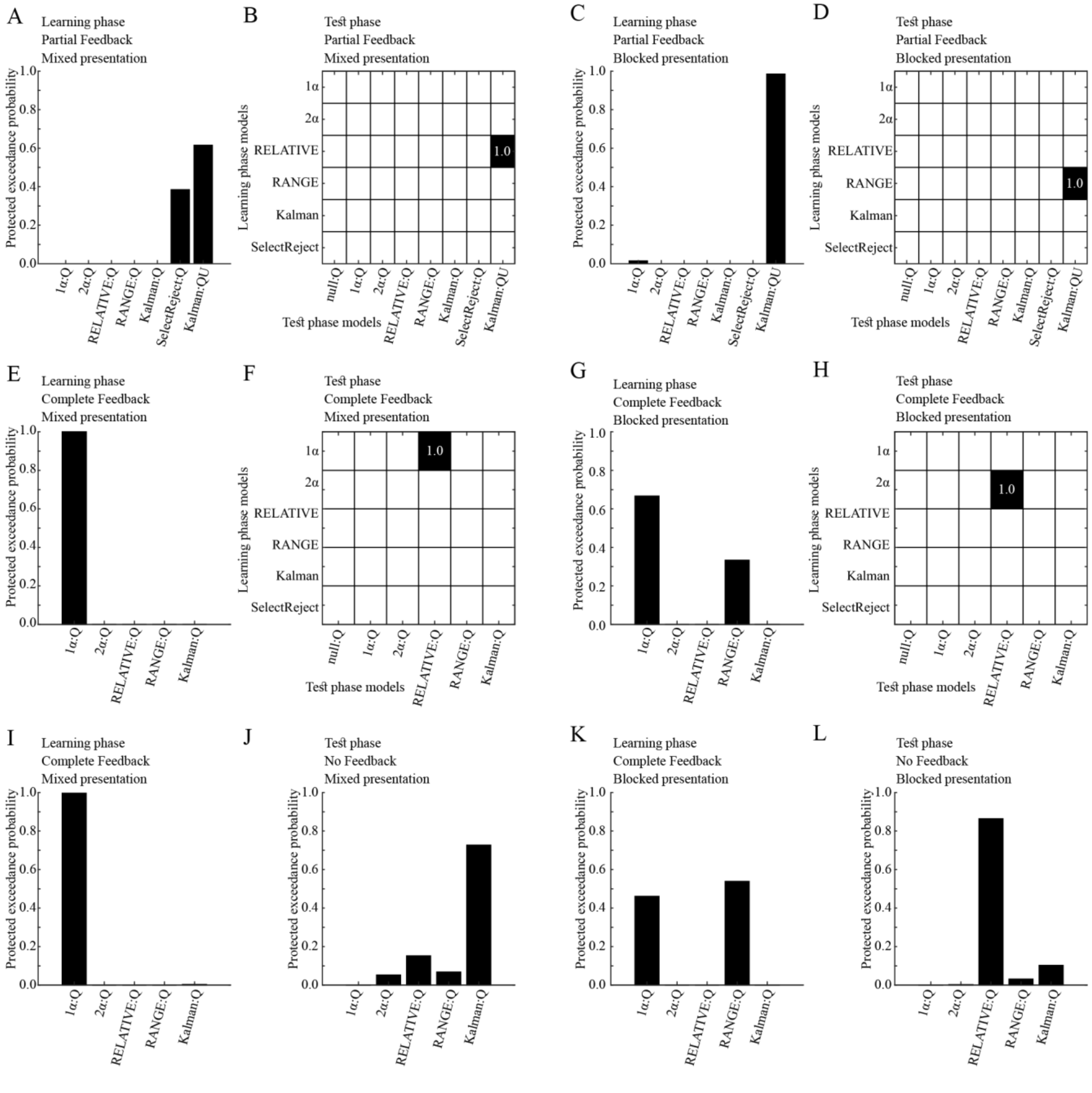

Supplementary Figure 2. Sample size n=50. **A.** Protected exceedance probabilities for the learning phase models when stimuli were presented in an inter-mixed fashion and with partial feedback. **B.** Protected exceedance probabilities for the test phase models when stimuli were presented in an inter-mixed fashion and with partial feedback. **C.** Protected exceedance probabilities for the learning phase models when stimuli were presented in a blockwise fashion and with partial feedback. **D.** Protected exceedance probabilities for the test phase models when stimuli were presented in a blockwise fashion and with partial feedback. **E.** Protected exceedance probabilities for the learning phase models when stimuli were presented in an inter-mixed fashion and with complete feedback. **F.** Protected exceedance probabilities for the test phase models when stimuli were presented in an inter-mixed fashion

and with complete feedback. **G.** Protected exceedance probabilities for the learning phase models when stimuli were presented in a blockwise fashion and with complete feedback. **H.** Protected exceedance probabilities for the test phase models when stimuli were presented in a blockwise fashion and with complete feedback. **I.** Protected exceedance probabilities for the learning phase models when stimuli were presented in an inter-mixed fashion and with complete feedback. **J.** Protected exceedance probabilities for the test phase models when stimuli were presented in an inter-mixed fashion and with no feedback. **K.** Protected exceedance probabilities for the learning phase models when stimuli were presented in a blockwise fashion and with complete feedback. **L.** Protected exceedance probabilities for the test phase models when stimuli were presented in a blockwise fashion and with no feedback. The X:Y denotation indicates Learning model:Decision model.

References

1. Bavard S, Rustichini A, Palminteri S. Two sides of the same coin: Beneficial and detrimental consequences of range adaptation in human reinforcement learning. *Science advances* **7**, (2021).
