# Supplementary material for: Estimation-uncertainty affects decisions with and without learning opportunities
Source: Nat Commun. 2025 Jul 21;16:6706. doi: 10.1038/s41467-025-61960-2 (PMC12280070; doi:10.1038/s41467-025-61960-2)
Supplement: Supplementary file 2 — Reporting Summary [file 41467_2025_61960_MOESM2_ESM.pdf]

nature portfolio

Corresponding author(s): Kristoffer C. Aberg

Last updated by author(s): Jun 20, 2025

Reporting Summary

Nature Portfolio wishes to improve the reproducibility of the work that we publish. This form provides structure for consistency and transparency in reporting. For further information on Nature Portfolio policies, see our Editorial Policies and the Editorial Policy Checklist.

Statistics

For all statistical analyses, confirm that the following items are present in the figure legend, table legend, main text, or Methods section.

n/a	Confirmed	
☐	☒	The exact sample size (*n*) for each experimental group/condition, given as a discrete number and unit of measurement
☐	☒	A statement on whether measurements were taken from distinct samples or whether the same sample was measured repeatedly
☐	☒	The statistical test(s) used AND whether they are one- or two-sided<br>*Only common tests should be described solely by name; describe more complex techniques in the Methods section.*
☐	☒	A description of all covariates tested
☐	☒	A description of any assumptions or corrections, such as tests of normality and adjustment for multiple comparisons
☐	☒	A full description of the statistical parameters including central tendency (e.g. means) or other basic estimates (e.g. regression coefficient) AND variation (e.g. standard deviation) or associated estimates of uncertainty (e.g. confidence intervals)
☐	☒	For null hypothesis testing, the test statistic (e.g. *F*, *t*, *r*) with confidence intervals, effect sizes, degrees of freedom and *P* value noted<br>*Give P values as exact values whenever suitable.*
☐	☒	For Bayesian analysis, information on the choice of priors and Markov chain Monte Carlo settings
☐	☒	For hierarchical and complex designs, identification of the appropriate level for tests and full reporting of outcomes
☐	☒	Estimates of effect sizes (e.g. Cohen's *d*, Pearson's *r*), indicating how they were calculated

Our web collection on statistics for biologists contains articles on many of the points above.

Software and code

Policy information about availability of computer code

Data collection	Data in the main experiment was collected using Matlab (v. 2022a) and the Psychophysics-3 toolbox (v. 3.0.19).
Data analysis	Data was analyzed using Matlab (v. 2022a) with the Statistics and Machine Learning Toolbox (v. 12.3) and the Computational and Behavioral Modeling toolbox (github.com/payampiray/cbm).

For manuscripts utilizing custom algorithms or software that are central to the research but not yet described in published literature, software must be made available to editors and reviewers. We strongly encourage code deposition in a community repository (e.g. GitHub). See the Nature Portfolio guidelines for submitting code & software for further information.

Data

Policy information about availability of data

All manuscripts must include a data availability statement. This statement should provide the following information, where applicable:

- Accession codes, unique identifiers, or web links for publicly available datasets
- A description of any restrictions on data availability
- For clinical datasets or third party data, please ensure that the statement adheres to our policy

The data from the main experiment is publicly available in an Open Science Framework repository 51, https://osf.io/3dk95/files/osfstorage. Data from the two complementary analyses are available in a Github repository 15, https://github.com/hrl-team/range.

Research involving human participants, their data, or biological material

Policy information about studies with human participants or human data. See also policy information about sex, gender (identity/presentation), and sexual orientation and race, ethnicity and racism.

Reporting on sex and gender	We had no hypotheses regarding the impact of sex or gender on behavior in our study, and these factors were therefore not considered in our analysis. Self-reported sex is reported.
Reporting on race, ethnicity, or other socially relevant groupings	We did not collect any data regarding race or ethnicity.
Population characteristics	See above
Recruitment	Participants were recruited from online advertisements (e.g. facebook student/campus groups) and by word-of-mouth.
Ethics oversight	The study was performed in accordance with the Declaration of Helsinki and approved by the ethical review board at the Weizmann Institute of Science (IRB protocol: 1086-2).

Note that full information on the approval of the study protocol must also be provided in the manuscript.

Field-specific reporting

Please select the one below that is the best fit for your research. If you are not sure, read the appropriate sections before making your selection.

☐ Life sciences ☒ Behavioural & social sciences ☐ Ecological, evolutionary & environmental sciences

For a reference copy of the document with all sections, see nature.com/documents/nr-reporting-summary-flat.pdf

Behavioural & social sciences study design

All studies must disclose on these points even when the disclosure is negative.

Study description	All data are quantitative, i.e. behavioral performance on a computerized task.
Research sample	Main experiment: 52 participants were recruited (27 male and 25 female; average age ± STD: 29.48±8.36), most being university students from the Weizmann Institute campus or from the neighboring Faculty of Agriculture.<br>The two additional datasets (copy/paste from their Method section): For the online experiments, we recruited 8 × 100 participants (414 females, aged 30.06 ± 10.10 years) from the Prolific platform (www.prolific.co).
Sampling strategy	A sample size calculation was not conducted for the main experiment. Instead, we replicate observed effects in two separate and independent datasets.
Data collection	In the main experiment, data collection was performed via a computerized task performed by participants in a dedicated experimental room. The experimenter was not in the room, nor could they influence the randomization of stimuli across conditions.
Timing	Main experiment: 13.07.2022-07.12.2022
Data exclusions	Two participants were excluded because their average performance in four learning conditions were below chance-level.
Non-participation	No participant dropped out
Randomization	NA

Reporting for specific materials, systems and methods

We require information from authors about some types of materials, experimental systems and methods used in many studies. Here, indicate whether each material, system or method listed is relevant to your study. If you are not sure if a list item applies to your research, read the appropriate section before selecting a response.

Materials & experimental systems

n/a	Involved in the study
☒	☐ Antibodies
☒	☐ Eukaryotic cell lines
☒	☐ Palaeontology and archaeology
☒	☐ Animals and other organisms
☒	☐ Clinical data
☒	☐ Dual use research of concern
☒	☐ Plants

Methods

n/a	Involved in the study
☒	☐ ChIP-seq
☒	☐ Flow cytometry
☒	☐ MRI-based neuroimaging

Plants

Seed stocks	NA
Novel plant genotypes	NA
Authentication	NA
